# Is Systemic Dissemination of BCG Following Neonatal Vaccination Required for Protection Against *Mycobacterium tuberculosis*?

**DOI:** 10.1093/infdis/jiaf051

**Published:** 2025-02-06

**Authors:** Tobias R Kollmann, Nelly Amenyogbe, Frederik Schaltz-Buchholzer, Ole Bæk, James Campbell, David J Lynn, Anita J Campbell, Peter Aaby, Christine Stabell Benn, Mihai G Netea, Maziar Divangahi

**Affiliations:** Canadian Center for Vaccinology, IWK, Dalhousie University, Halifax, Nova Scotia, Canada; Canadian Center for Vaccinology, IWK, Dalhousie University, Halifax, Nova Scotia, Canada; Bandim Health Project, INDEPTH Network, Bissau, Guinea-Bissau; Department of Clinical Research, University of Southern Denmark and Odense University Hospital, Copenhagen; Comparative Pediatrics and Nutrition; BRIDGE Translational Excellence Programme, Faculty of Health Sciences, University of Copenhagen, Frederiksberg, Denmark; Central Perth Skin Clinic, Perth, Western Australia, Australia; Precision Medicine, South Australian Health and Medical Research Institute, Adelaide, South Australia; Flinders Health and Medical Research Institute, Flinders University, Bedford Park, South Australia; Infectious Diseases Department, Perth Children's Hospital, Perth; Kids Research Institute Australia, Wesfarmers Centre of Vaccines and Infectious Diseases, University of Western Australia, Perth; Department of Infectious Diseases, Perth Children's Hospital, Nedlands, Western Australia, Australia; Bandim Health Project, INDEPTH Network, Bissau, Guinea-Bissau; Bandim Health Project, Odense Patient Data Explorative Network, Department of Clinical Research, Odense University Hospital; Danish Institute for Advanced Study, University of Southern Denmark, Copenhagen, Denmark; Department of Internal Medicine and Infectious Diseases, Radboud University Medical Center, Nijmegen, The Netherlands; Life and Medical Sciences Institute, Department of Immunology and Metabolism, University of Bonn, Germany; Departments of Medicine, Pathology, and Microbiology & Immunology, McGill University Health Centre, McGill International Tuberculosis Centre, Meakins Christie Laboratories, McGill University, Montreal, Quebec, Canada

**Keywords:** TB, BCG, bacteremia, neonate, adult

## Abstract

Tuberculosis (TB) is caused by *Mycobacterium tuberculosis* (*Mtb*) and is a leading cause of death. BCG is the only licensed TB vaccine. Preclinical studies have shown that in adults, intravenous administration of BCG improves protection against TB. We hypothesize that intradermal administration of BCG to the human newborn leads to low-grade BCG bacteremia and that this systemic dissemination improves protection against *Mtb* infection. This hypothesis is based on supporting observations including animal and human studies. It is a testable hypothesis and offers to deliver immediately actionable insight to advance the global efforts against TB.

## Teaser

Intradermal BCG does not sufficiently protect adults from *Mycobacterium tuberculosis*; in preclinical models, adult protection improves if BCG is administered intravenously. But newborn intradermal BCG leads to BCG bacteremia and potently protects. Therefore, BCG bacteremia could be a correlate of protection.

## Graphical Abstract

**Graphical Abstract:**
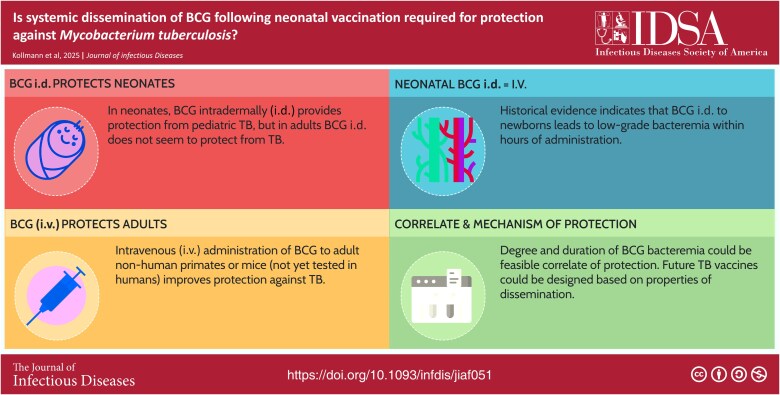
This graphical abstract is also available at Tidbit: https://tidbitapp.io/tidbits/is-systemic-dissemination-of-bcg-following-neonatal-vaccination-required-for-protection-against-mtuberculosis/update

Lack of insight into mechanisms leading to prevention of *Mycobacterium tuberculosis* (*Mtb*) infection or progression to tuberculosis (TB) disease hinders the development of effective vaccines [1, 2]. Recent preclinical studies have shown that intravenous (i.v.) administration of BCG vaccine improves protection from infection with *Mtb* [3–7]. We hypothesize that neonatal intradermal (i.d.) BCG administration leads to low-grade bacteremia in newborns and that this systemic spread, similar to adult i.v. administration, improves protection from *Mtb* infection via “early clearance.” Our hypothesis is supported by several observations. First, in adult animal models (including nonhuman primates), BCG-induced protection from infection with *Mtb* is better if BCG is given intravenously (i.v.) as compared with intradermal (i.d.) or subcutaneous routes [3–7]. Second, BCG vaccination of newborns can systemically disseminate [8–10]. Third, Neonatal administration of BCG provides protection from both pulmonary and especially extrapulmonary TB, yet there is no consistent evidence of protection in adults; that is, BCG administered i.d. to adults leads to protection from *Mtb* infection of adult recipients only in some settings and populations [1, 2]. Variation in protection following BCG i.d. in adults may in fact relate to variation in BCG bacteremia across settings and populations. Last, BCG vaccination, especially of newborns, enhances “early clearance” of *Mtb* [6, 11–13]. Early clearance denotes the observation that exposure to *Mtb* does not lead to infection because *Mtb* can be eradicated before an infection is established—that is, the interferon-γ release assay remains negative despite *Mtb* exposure [11, 13, 14].

Each component of this hypothesis is testable:

1. Systemic spread in newborns: Assess blood samples for the presence of BCG (bacteremia) following BCG administration to human newborns. We anticipate that detection of BCG bacteremia would be rare using blood cultures and will require more sensitive molecular assays; this assumption is based on the fact that several large studies have shown neonatal BCG to not lead to clinically symptomatic bacteremia [15–18]. There have only been 4 cases of newborns who became clinically symptomatic (fever, jaundice) within 48 hours of receiving BCG i.d. and were found to be BCG bacteremic; these were all reported from Thailand following administration by the same nurse of a 0.1-mL dose (ie, twice the standard neonatal dose, as is routine in Thailand) [10, 19].
2. Age dependence of systemic spread: Assess blood samples as above following i.d. administration in different age groups [20].
3. Early clearance following systemic spread: Assess whether systemic dissemination is associated with “early clearance,” that is, epigenetic and metabolic reprogramming of immune cells following BCG vaccination (a process also termed “trained immunity”), which can readily be done using existing standardized assays on peripheral blood samples [1]. Finally, correlating the immune changes with degree and duration of systemic BCG dissemination across age would firmly establish an association; such association could then be investigated for causality in animal models [3–5].

Testing this hypothesis would deliver the following actionable insights and outcomes:

1. Insight into mechanisms: Insight into the molecular mechanisms that allow BCG to enhance “early clearance” would directly support the design of future TB vaccines based on the fundamental properties of systemic dissemination, that is, the ability to induce memory in both adaptive and innate immune compartments [1, 21]. Furthermore, if BCG i.d. in newborns acts similar to BCG i.v. in adults, then assessing ways to enhance systemic spread in adults to mimic the newborn dissemination, but without the need for i.v. administration, could provide alternate avenues to improve adult BCG-induced protection from infection with *Mtb* [22]. This could, for example, be achieved by increasing local blood flow in the skin following BCG administered i.d. [23].
2. Insight into correlates of protection: Degree and duration of systemic BCG dissemination following human newborn i.d. administration could be evaluated as a feasible correlate of protection. Concretely, quantifiable BCG bacteremia could be a proxy for BCG entering the bone marrow, where it gains access to hematopoietic stem cells (HSCs) and induces changes in the transcriptional landscape of HSCs and the myeloid cells subsequently derived from them. Innate myeloid cells generated from such BCG-trained HSCs have been shown to exhibit superior clearance of *Mtb* [1, 6]. Potentially, dissemination could also lead to stronger adaptive immune responses.
3. Insight into policy of delivery of BCG: Given that epithelial skin barriers change rapidly early in life, any delay in administering BCG immediately at birth, even by a few days, may reduce propensity for systemic spread and with that the protective benefit [20]. While the World Health Organization currently recommends that BCG be given at birth, <50% of newborns in the world receive BCG prior to the end of their first month of life [24]. The reasons for this delay are multifactorial yet include restrictive vial-opening policies that could readily be changed [25]. The data generated through testing this hypothesis could add an incentive to administer BCG at birth to all eligible newborns.

In summary, investigating the outlined hypothesis could enhance the impact of the existing BCG vaccine and guide the development of future TB vaccines.

## Notes

***Author contributions.*** All authors substantially contributed to the conception and design of this manuscript and drafted the work, reviewed it critically, approved the final version, and agree to be accountable for all aspects of the work presented.

***Financial support.*** T. R. K. is recipient of funding from the National Institutes of Health/National Institute of Allergy and Infectious Diseases (grant numbers U19AI118608 and U19AI168643), Open Philanthropy, and Dalhousie University Faculty of Medicine and Medical Research Development Office. N. A. is supported by the Canadian Institutes of Health Research REDI Fellowship (503114). O. B. is supported by the BRIDGE–Translational Excellence Programme (bridge.ku.dk) at the Faculty of Health and Medical Sciences, University of Copenhagen, funded by the Novo Nordisk Foundation (grant agreement numbers NNF23SA0087869 and NNF20SA00643). D. J. L. is supported by the Australian National Health and Medical Research Council and the South Australian Department of Health. A. J. C. is supported by a Raine Clinician Research Fellowship. M. D. is supported by the CIHR (project grant number 168885), the NIH (grant number U01-AI160406), and the Gates Foundation (grant number INV-003360).
